# Parental Family Structure, Helicobacter Pylori, and Gastric Adenocarcinoma

**DOI:** 10.1371/journal.pmed.0040025

**Published:** 2007-01-16

**Authors:** Pagona Lagiou, Dimitrios Trichopoulos

The study of family of origin (parental family), as contrasted to family of procreation, originally received attention from epidemiology in the context of childhood infectious diseases. Two aspects of parental family have been intensely investigated: sibship size and birth order [1,2]. Larger sibship size increases the likelihood of introduction and spread of infectious agents within the family and tends to be inversely associated with children's average age at infection. Birth order, in the absence of vaccination, has a more specific effect on average age at infection, since first-born children are usually infected when first exposed to the child care or school environment, whereas later-born children tend to be infected earlier, even in utero, by their older siblings.

## Parental Family and Helicobacter Pylori

Larger sibship size and higher birth order have long been known to be associated with higher prevalence of chronic infections by the hepatitis B virus [3]. More recently, similar associations have been reported for infection with Helicobacter pylori [4]. Clearly, since these microorganisms are etiologically related to hepatocellular carcinoma and gastric adenocarcinoma, respectively, sibship size and birth order should be—and have been reported to be [5,6]—risk factors for these malignancies.

A more subtle question, however, is the following: given chronic carrier state, does larger sibship size and higher birth order, as indicators of earlier infection, predict higher risk of cancer at the corresponding site? For hepatitis B virus and hepatocellular carcinoma this question has been affirmatively answered [7], and the new study by Blaser and colleagues published in *PLoS Medicine* goes a long way in documenting a similar association for H. pylori and gastric adenocarcinoma [8].

## The New Study

In a cohort of 9,935 Japanese-American men, who had provided blood samples at entry in the study and were followed for 28 years, 261 cases of non-cardia gastric cancer were identified. In a nested case-control design, each case patient was paired with one control, matched for age at examination and date of serum collection. Of the 261 patients with non-cardia gastricH. pyloriEarlier age at establishment of H. pylori carrier state increases the risk of gastric cancer decades later. cancer, 239 (92%) were carriers of H. pylori on the basis of antibody status and 189 (72%) carried *cagA^+^* strains, which are considered more virulent. The corresponding numbers and proportions among the 261 matched controls were 205 (79%) and 155 (59%). In line with the study objective, further analyses were restricted to patients who were serologically positive for H. pylori in general or *cagA^+^* in particular cases and controls.

Gastric cancer cases were further distinguished, on the basis of Lauren's classification [9], into the more common intestinal type, the less common diffuse type, and the uncommon mixed, other, and unknown types. Among those who were H. pylori positive, there was a significant positive association of gastric adenocarcinoma with sibship size, which was somewhat more evident for the diffuse type. With respect to birth order, a borderline significant positive association was only evident for the intestinal type. Results for both sibship size and birth order were generally more striking when analyses were limited to *cagA^+^* study participants. The results of the study by Blaser and colleagues [8] strongly indicate that earlier age at establishment of H. pylori carrier state increases the risk of gastric cancer several decades later. There are several plausible explanations considered by the authors, but the empirical evidence implicating early intra-familial transmission points to intra-familial selection of better adapted and more virulent strains [10] in a background of age-modulated immune response.

## Strengths and Limitations of the Study

Blaser and colleagues [8] have used an important cohort to evaluate evolving concepts on the natural history of H. pylori infection and have reached conclusions that are of both theoretical and practical importance. A limitation of their study is the relatively small number of cases with gastric adenocarcinoma but, clearly, not much could be done about this. Another limitation is that mutual control of sibship size and birth order was not attempted. These two variables are strongly positively correlated (one cannot have a high birth order unless one belongs in a large sibship) and association of gastric cancer with just one of them would be reflected in an association with the other as well. In this instance, however, since increases in both of these variables tend to reduce age at infection, the reported associations point to a biologically sound and valuable conclusion.

Whether sibship size or birth order is the driving force in the association of early age at establishment of chronic H. pylori infection with gastric adenocarcinoma, socioeconomic status plays a critical role. This is because in most settings low socioeconomic status is associated with larger sibship size and modulates the consequences of birth order by intensifying the intra-familial transmission of contagious agents. The results of the investigation by Blaser and colleagues, however, allow a better insight into past trends of gastric cancer incidence and may improve predictions of future trends by introducing into the equation two additional, albeit interrelated, explanatory variables.

## Next Steps

A broader lesson from this study is that age at infection or establishment of chronic carrier state is a variable of considerable importance in assessing the carcinogenic potential of infectious agents. These consequences are unlikely to be uniform across the various cancer types—we already have evidence that late infection with unspecified agent(s) increases the risk of childhood leukemia [11,12]. Identifying the agents' molecular characteristics or the hosts' immunological aspects in early or late infection is a field that merits further attention in the study of cancer etiology.
